# Impact of Enzymatically Extracted High Molecular Weight Fucoidan on Lipopolysaccharide-Induced Endothelial Activation and Leukocyte Adhesion

**DOI:** 10.3390/md21060339

**Published:** 2023-05-31

**Authors:** Nora Kirsten, Julia Ohmes, Maria Dalgaard Mikkelsen, Thuan Thi Nguyen, Martina Blümel, Fanlu Wang, Deniz Tasdemir, Andreas Seekamp, Anne S. Meyer, Sabine Fuchs

**Affiliations:** 1 Experimental Trauma Surgery, University Medical Center Schleswig-Holstein, 24105 Kiel, Germany; 2 Protein Chemistry and Enzyme Technology Section, DTU Bioengineering, Department of Biotechnology and Biomedicine, Technical University of Denmark, 2800 Kongens Lyngby, Denmark; mdami@dtu.dk (M.D.M.);; 3 GEOMAR Centre for Marine Biotechnology (GEOMAR-Biotech), Research Unit Marine Natural Products Chemistry, GEOMAR Helmholtz Centre for Ocean Research Kiel, 24106 Kiel, Germany; 4 Faculty of Mathematics and Natural Science, Kiel University, 24118 Kiel, Germany

**Keywords:** fucoidan, *Fucus evanescens*, HMWF, outgrowth endothelial cells, inflammation, monocytes, lipopolysaccharide

## Abstract

The endothelial cell lining creates an interface between circulating blood and adjoining tissue and forms one of the most critical barriers and targets for therapeutical intervention. Recent studies suggest that fucoidans, sulfated and fucose-rich polysaccharides from brown seaweed, show multiple promising biological effects, including anti-inflammatory properties. However, their biological activity is determined by chemical characteristics such as molecular weight, sulfation degree, and molecular structure, which vary depending on the source, species, and harvesting and isolation method. In this study, we investigated the impact of high molecular weight (HMW) fucoidan extract on endothelial cell activation and interaction with primary monocytes (MNCs) in lipopolysaccharide (LPS)-induced inflammation. Gentle enzyme-assisted extraction combined with fractionation by ion exchange chromatography resulted in well-defined and pure fucoidan fractions. FE_F3, with a molecular weight ranging from 110 to 800 kDa and a sulfate content of 39%, was chosen for further investigation of its anti-inflammatory potential. We observed that along with higher purity of fucoidan fractions, the inflammatory response in endothelial mono- and co-cultures with MNCs was reduced in a dose-dependent manner when testing two different concentrations. This was demonstrated by a decrease in IL-6 and ICAM-1 on gene and protein levels and a reduced gene expression of TLR-4, GSK3β and NF-kB. Expression of selectins and, consequently, the adhesion of monocytes to the endothelial monolayer was reduced after fucoidan treatment. These data indicate that the anti-inflammatory effect of fucoidans increases with their purity and suggest that fucoidans might be useful in limiting the inflammatory response of endothelial cells in cases of LPS-induced bacterial infection.

## 1. Introduction

Inflammation is a complex biological response to harmful stimuli and a crucial host defense mechanism of innate immunity. It is now well established that the endothelium, as the barrier between circulating blood and the adjacent tissue, serves as a major mediator of the inflammatory process [1,2]. Whether in local infections, burn injuries, or ischemic events, endothelial activation is a critical determent during the development of a variety of diseases. However, imbalanced inflammatory reactions can turn an effective host defense mechanism into an injurious insult and may lead to endothelial dysfunction, such as chronic inflammation, atherosclerosis [3], or septic shock [4,5,6]. This underlines the role of the endothelium as an important therapeutic target to limit inflammatory reactions [7].

Lipopolysaccharide (LPS), a key component of the outer membrane of gram-negative bacteria, is a particularly potent activator of endothelial cells. The binding of LPS to the pattern recognition receptor toll-like receptor (TLR) 4 on the endothelial surface [8,9] initiates a downstream signaling cascade resulting in the activation of the nuclear factor-kB (NF-kB), mitogen-activated protein kinase (MAPK) and phosphatidylinositol 3-kinase (PI3K)/Akt pathways [10]. Activation can be further modified by glycogen synthase kinase 3 β (GSK-3β) [11,12], which then leads to the expression of pro-inflammatory proteins. Among these are cytokines, such as interleukin-6 (IL-6) and tumor necrosis factor-alpha (TNF-α), chemokines and adhesion molecules, such as E-Selectin, intercellular adhesion molecule (ICAM-1) and vascular adhesion molecule (VCAM-1), that bind leukocytes to allow their emigration from circulating blood into the tissue [13]. The cytokines produced during inflammatory processes, including IL-6, act as major regulators of the inflammatory response by inducing the production of acute-phase proteins, increasing vascular permeability [14], and recruiting leukocytes to the inflammatory site [15].

Fucoidans, sulfated and fucose-rich polysaccharides from brown seaweed, are gaining interest as a marine compound due to their broad spectrum of bioactivity. It was shown that fucoidans act on coagulation [16], angiogenesis [17,18], osteogenesis [19], and inflammation [20,21,22]. Despite these promising biological effects, the biomedical application of fucoidans remains limited due to their varying chemical structure and, consequently, their highly variable bioactivity [23]. Decisive factors that influence the monosaccharide composition and sulfate content include seaweed species, growing conditions, harvesting time, extraction, and purification method [24,25]. This results in heterogeneous chemical properties explaining the inconsistent findings for the biological mode of action of different fucoidan extracts. The implication is the need for chemically and biologically characterized extracts obtained via standardized and reproducible extraction methods.

In this study, we investigated the effect of high molecular weight fucoidan fractions with increasing purity (FE_FE1, FE_FE2, FE_F3) on endothelial activation in vitro and compared it with a commercially available crude extract of *Fucus vesiculosus* (FV_crude). The fractions were obtained via enzyme-assisted extraction from *Fucus distichus* subsp. *evanescens* (FE) and further purified using ion-exchange chromatography (IEX) and studied for structural and molecular properties as previously described in [26]. The anti-inflammatory activity was measured by applying mono-cultures of primary microvascular endothelial cells and co-culture models with monocytes. Both cell types were isolated from human blood to provide an experimental model for its application as an anti-inflammatory agent.

## 2. Results

In this study, we used FE_crude isolated from *Fucus distichus* subsp. *evanescens* by an enzymatic-assisted extraction process and corresponding fractions FE_F1, FE_F2, FE_F3 gained by IEX (ion exchange chromatography) fractionation. The molecular weight and the monosaccharide composition, including the fucose content, were previously described and published in detail by Nguyen et al. (2020) [26]. The fucose and sulfate content in% increased from FE_crude (24.8 ± 2.9 /21.7 ± 0.5) before IEX to FE_F1 (34 ± 3.1/20.4 ± 3.4), FE_F2 (74.7 ± 0.8/34.8 ± 2.0) and finally FE_F3 (87.8 ± 1.4/38.7 ± 1.0) resembling the purest fraction in terms of the fucose content after this purification step. The extract FE_crude and all fractions resulting from IEX can be considered high molecular weight fucoidans with only minor variations in size distribution. The main molecular weight peak is 400 kDa for FE_crude and FE_F1 and 600 kDa for FE_F2 and FE_F3 [18]. Further, commercially obtained crude HMWF from *Fucus vesiculosus* (Sigma-Aldrich) was used as an internal reference for an unpurified fucoidan with an impact on endothelial cells and vascularization. According to the manufacturer’s analysis, the molecular weight of this extract is 135 kDa, and the sulfate content is 9%.

### 2.1. Influence of Fucoidan Extracts on Inflammatory Mediators in OECs

**Figure 1:**
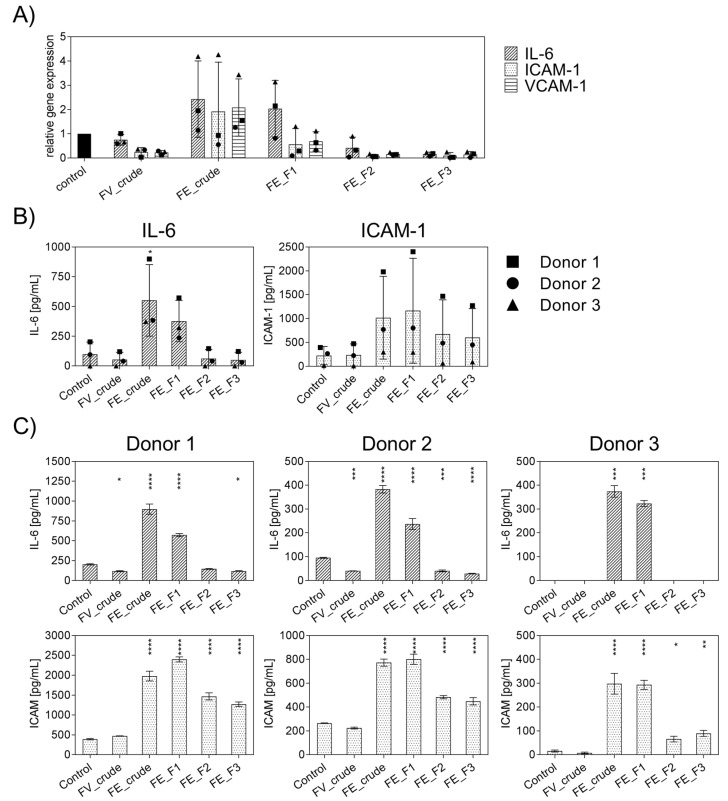
Effect of different fucoidan extracts on gene and protein levels of the pro-inflammatory cytokine IL-6 and adhesion molecules ICAM-1 and VCAM-1 in outgrowth endothelial cells (OECs). (**A**) Gene expression and (**B**) protein level were quantified after seven days of treatment with 100 μg/mL fucoidan by qPCR and ELISA, respectively. The mean values of experiments with three individual donors ± SD were plotted. (**C**) Protein levels of IL-6 and ICAM-1 are depicted for individual donors ± SD. Significance compared to the control was calculated with the One-way ANOVA (* *p* < 0.05, ** *p* < 0.01, *** *p* < 0.001, **** *p* < 0.0001). *n* = 3.

Firstly, the general effect of the enzymatically extracted FE fractions on endothelial activation was tested to select suitable fractions with a low inflammatory profile and to determine the impact of fucoidan extracts with different purities or from different sources on inflammatory mediators. In these experiments, inflammation was not actively induced, and outgrowth endothelial cells (OECs) were treated with the different fractions of FE extract and control extracts for only seven days, and the gene expression levels of IL-6, ICAM-1, VCAM-1 (Figure 1A) were quantified. Additionally, the protein levels of IL-6 and ICAM-1 were determined and depicted in Figure 1B,C. For comparison, we performed the experiment with an established, commercially available extract, FV_crude (Sigma Aldrich), as an additional reference for an unpurified fucoidan with a high molecular weight. Assays were performed as previously described to ensure that the tested doses of fucoidan were non-toxic to OECs, MTS, and LDH [18]. The influence of endotoxins, potentially contained in the extracts, on endothelial cell activation was ruled out via EndoLISA. Treatment with FE_crude and FE_F1 resulted in inflammatory activation of OECs, which was indicated by an increase in IL-6 and ICAM-1 compared to untreated controls (Figure 1A). However, treatment with the purer fractions FE_F2 and FE_F3 decreased the mRNA levels of IL-6, ICAM-1, and VCAM-1 almost completely (Figure 1A). Protein levels of IL-6 for purer fractions remained similar to the untreated control (Figure 1B). For ICAM-1, treatment with all fractions resulted in elevated protein levels. However, treatment with the purer fractions (FE_F2, FE_F3) leads to a lower increase in ICAM-1. This experiment demonstrates that the purer fucoidan fractions FE_F2 and FE_F3 per se did not cause inflammatory activation of endothelial cells, as demonstrated for several donors (Figure 1C). FE_F3 was found to be the fraction with the most promising effect and highest purity and, thus, selected for further experiments.

### 2.2. Effect of Fucoidan Extracts on Rna Expression of Markers for Endothelial Activation in LPS-Stimulated OECs

**Figure 2:**
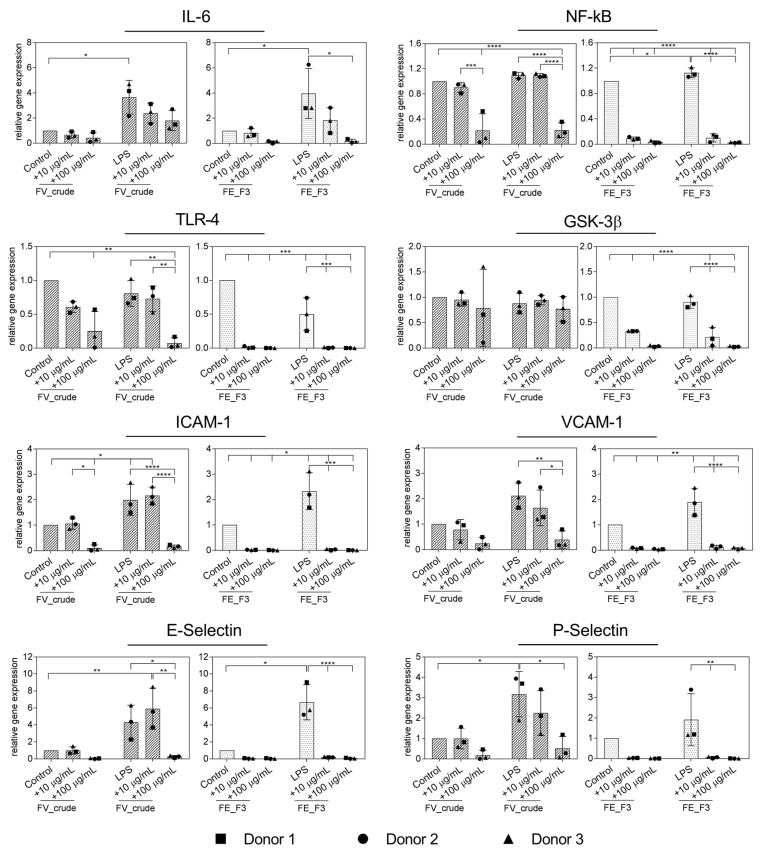
Effect of fucoidan extracts FV_crude and FE_F3 on the RNA expression of pro-inflammatory markers of the innate immune response and adhesion molecules in OECs. OECs were treated with 10 μg/mL and 100 μg/mL fucoidan for five days. After 48 h of stimulation with 100 ng/mL lipopolysaccharide (LPS), gene expression was quantified by qPCR. The mean values of experiments with three individual donors ± SD were plotted. Significance was calculated with One-way ANOVA (* *p* < 0.05, ** *p* < 0.01, *** *p* < 0.001, **** *p* < 0.0001).

In the next step, inflammation was actively induced in endothelial cells by treatment with LPS. In order to examine the impact of fucoidan on the inflammatory response of LPS-stimulated OECs, we quantified the expression of the inflammatory mediators IL-6, NF-kB, TLR4, GSK3β and adhesion molecules ICAM-1, VCAM-1, E-Selectin and P-Selectin (Figure 2). OECs were treated with the fucoidan extracts in different doses for five days, followed by two days of LPS stimulation, while fucoidan treatment continued. The expression of IL-6 was down-regulated after FE_F3 treatment, while treatment with LPS alone markedly increased the IL-6 expression in OECs compared to the untreated control cells. Both extracts, FV_crude and FE_F3, decreased the gene levels of NF-kB and TLR4 in LPS-stimulated OECs. However, FV_crude required a higher concentration to show a significant effect. FE_F3 reduced the expression of GSK3β almost completely, whereas FV_crude did not affect the GSK3β expression. Treatment with LPS resulted in a remarkable up-regulation of the expression of the adhesion molecules ICAM-1, VCAM-1, E-Selectin, and P-Selectin. Both tested extracts lowered the expression of those markers in a dose-dependent manner, although FE_F3 reduced the gene levels of the adhesion molecules to a higher extent. The results indicate that pre-treatment with FV_crude and FE_F3 may reduce the LPS-mediated endothelial activation in OEC mono-cultures.

### 2.3. Effect of Fucoidan Extracts on Endothelial Activation in LPS-Stimulated OEC

**Figure 3:**
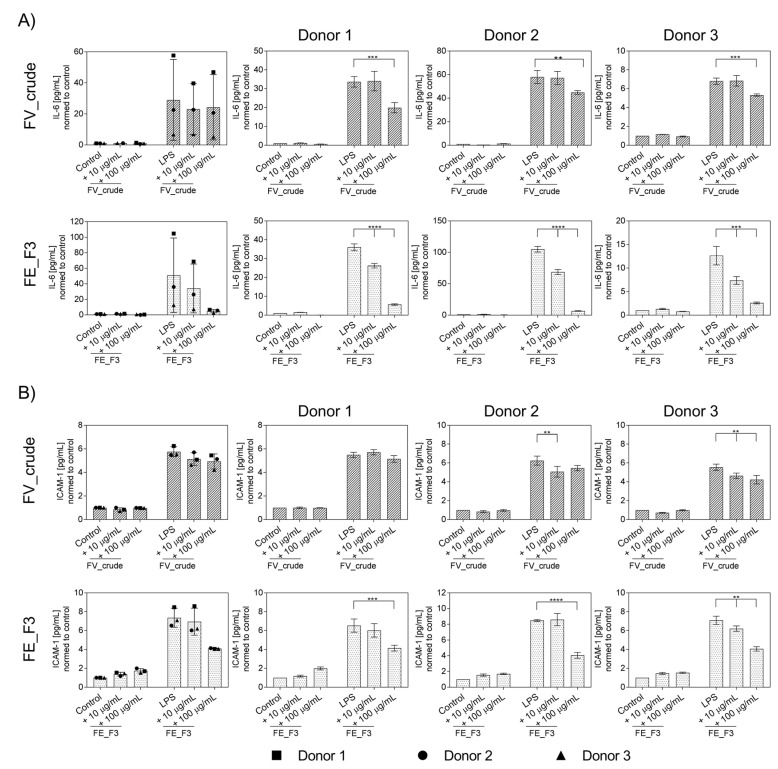
Effect of fucoidan extracts FV_crude and FE_F3 on the protein level of pro-inflammatory cytokine IL-6 (**A**) and ICAM-1 (**B**) in OECs. OECs were treated with 10 μg/mL and 100 μg/mL fucoidan for five days. After 48 h of stimulation with 100 ng/mL LPS, protein levels were quantified using ELISA and normalized to the control. The mean values of experiments with three individual donors ± SD were plotted. Significance was calculated with One-way ANOVA (** *p* < 0.01, *** *p* < 0.001, **** *p* < 0.0001). *n* = 3.

To further determine the influence of fucoidan extracts on the inflammatory activation of OECs, protein levels of IL-6 (Figure 3A) and ICAM-1 (Figure 3B) were quantified from the supernatants using ELISA for several donors of endothelial cells. OECs were treated with fucoidan for five days, followed by two days of LPS stimulation. Treatment with LPS resulted in a markable up-regulation in the levels of IL-6 and ICAM-1. Fucoidan pre-treatment reduced the secretion of the markers dose-dependently. This effect becomes clearer when the donors are considered individually to exclude the effects of donor-to-donor variation. FE_F3 showed a significant reduction in IL-6 at both tested concentrations of 10 μg/mL and 100 μg/mL. FV_crude reduced the IL-6 secretion only at the higher concentration of 100 μg/mL (Figure 3A). Protein levels of ICAM-1 were down-regulated after treatment with 100 μg/mL FE_F3 (Figure 3B). These results demonstrate that the tested extracts decrease the secretion of key inflammatory mediators in OEC mono-cultures.

### 2.4. Impact of Fucoidan Extracts on the Adherence of Monocytes to the Endothelial Cell Layer

**Figure 4:**
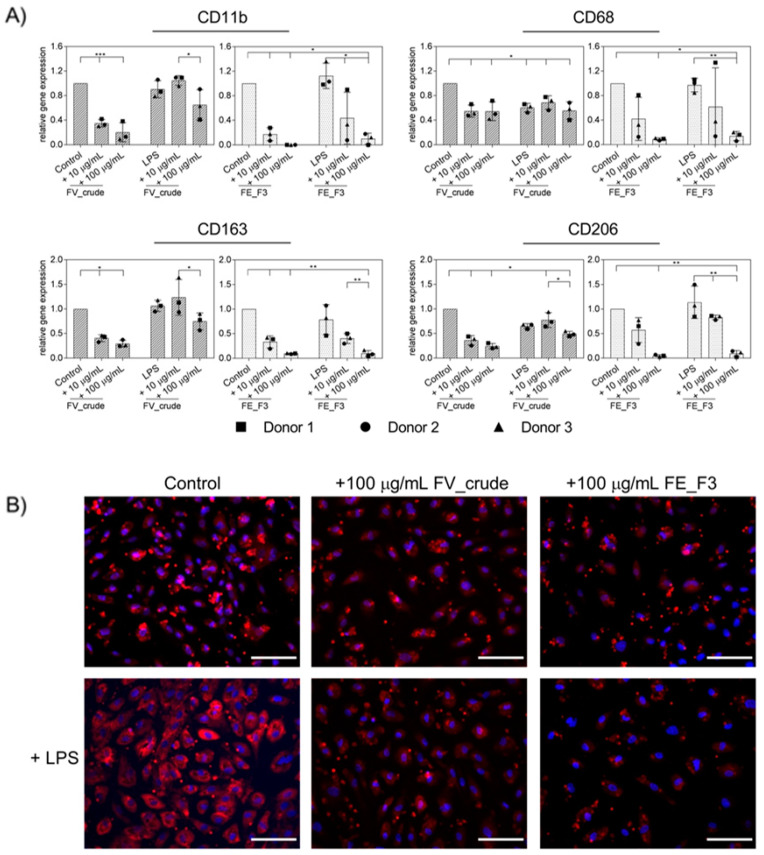
(**A**) Effect of fucoidan extracts FV_crude and FE_F3 on the expression of monocyte surface markers in OEC-monocyte-co-cultures. CD11b, CD68, CD163, and CD206 were determined as indicators for the adhesion of monocytes on the OEC cell layer. OECs were treated with 10 μg/mL and 100 μg/mL fucoidan for five days. After 24 h of stimulation with 100 ng/mL LPS, monocytes were added, and fucoidan–LPS treatment continued for 48 h. Gene expression was quantified by qPCR. The mean values of experiments with three individual donors ± SD were plotted. Significance compared to the control was calculated with One-way ANOVA (* *p* < 0.05, ** *p* < 0.01, *** *p* < 0.001). *n* = 3. (**B**) The co-cultures were fixed and stained for CD68 and nuclei (CD68 in red, nuclei in blue, scale bars = 100 μm).

The transmigration of activated immune cells from the peripheral blood to the tissue plays a critical role in the state of inflammation. In order to examine the effect of the fucoidan extracts on the cell–cell interactions between OECs and monocytes, we applied it to an OEC monocyte co-culture system. OECs were treated with the fucoidan extracts for five days; on the sixth day, stimulation with 100 ng/mL LPS was initiated. Twenty-four hours after LPS treatment, primary monocytes isolated from endothelial progenitor cell cultures (EPCs) were added to the OEC monolayer, and treatment continued for two days. As an indicator for the binding of monocytes to the endothelial layer, we quantified the mRNA levels of the monocyte surface markers CD11b, CD68, CD163, and CD206 via qPCR (Figure 4A). Treatment with both extracts reduced the gene levels of the tested MNC-associated markers. The observed effect was strongest when cells were treated with FE_F3. Staining for CD68 was applied to visualize further the influence of the fucoidan fractions (Figure 4B). The expression of CD68 was elevated after LPS stimulation, treatment with FV_crude and FE_F3 reduced the expression. The experiment demonstrates that pre-treatment with fucoidan reduces the adherence of monocytes to the endothelial cell layer, thus functionally influencing endothelial/MNC interaction.

### 2.5. Influence of Fucoidan Extracts on the Adherence of Native Monocytes to the Endothelial Cell Layer

**Figure 5:**
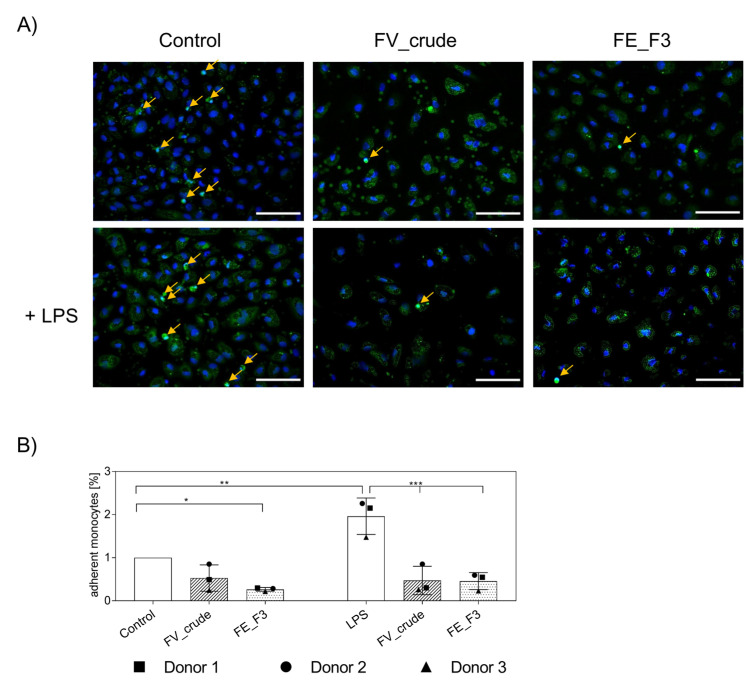
(**A**) Effect of fucoidan extracts FV_crude and FE_F3 on the adhesion of monocytes to the OEC-monolayers. OECs were treated with 100 μg/mL FV_crude and 100 μg/mL FE_F3 for five days. After 24 h of stimulation with 100 ng/mL LPS, monocytes were tagged with CellTracker^TM^ Green and added to the culture. Fucoidan–LPS treatment continued for four hours. These cells were fixed and stained for nuclei (nuclei in blue, monocytes in green, scale bars = 100 μm). (**B**) Adherent monocytes were counted and depicted in% compared to the control. The mean values of experiments with three individual donors ± SD were plotted. Significance compared to the control was calculated with One-way ANOVA (* *p* < 0.05, ** *p* < 0.01, *** *p* < 0.001).

After we observed a decrease in the adhesion of pre-cultured MNCs for fucoidan-treated samples, the experiment was repeated with native MNCs isolated directly from the blood. In these experiments, we found a similar decrease in adherent monocytes in OEC and monocyte co-cultures (Figure 5). For visualization, we labeled primary monocytes with CellTracker™ Green before adding them to the endothelial cell layer. After fixation, cells were stained for nuclei (Figure 5A). Vital monocytes, with distinct nuclear counterstain, were counted (Figure 5B) and depicted in% compared to the untreated control. LPS treatment induced an increase in adherent monocytes that was inhibited by FV_crude and FE_F3 treatment. When treated with FE_F3, levels of adherent monocytes were found below the untreated control.

## 3. Discussion

First extracted in 1913, fucoidans are known and investigated for their multifaceted bioactivity [27]. Despite various promising biological effects, their biomedical application remains limited due to strong structure–function dependence. Generally composed of a backbone of α(1→3)-l-fucopyranose residues or alternating α(1→3) and α(1→4)-linked l-fucopyranosyls [23], monosaccharide composition, branching, sulfation degree, and molecular weight may vary depending on seaweed species, environmental factors, harvesting time, and extraction and purification method [28,29,30]. These variations in fucoidan chemical structure result in occasionally contradictory findings regarding their bioactivity. In this study, we investigated the anti-inflammatory properties of a chemically characterized fucoidan extract from *Fucus distichus* subsp. *evanescens* in mono-cultures of outgrowth endothelial cells (OECs) and a co-culture model with monocytes. Fucoidan was extracted by an enzyme-assisted procedure and further fractionated using ion exchange chromatography as described by Nguyen and colleagues [26]. This yielded three fractions that consist of a backbone of 1→3 and 1→4 glycosidic linkages and differ mainly in fucose content and sulfation degree [18].

To compare and select the most suitable fraction, gene expression and protein levels of pro-inflammatory markers and adhesion molecules were measured in OECs first under a non-inflammatory state. The third eluted and purest extract, FE_F3, did not activate the endothelial cells or partly reduce the gene expression of inflammatory markers. This result shows the highest bioactivity of FE_F3, which is consistent with the findings by Ohmes et al. (2020) [18]. FE_F3, as the purest fraction with the highest sulfate and fucose content, showed the strongest anti-angiogenic effects in this previous study dealing with angiogenesis [18]. By decreasing Ang-2 levels, responsible for endothelial activation [31], FE_F3 counteracted angiogenesis and stabilized the endothelial barrier. Thus, FE_F3 has the potential to reduce not only angiogenesis [18] but also the inflammatory activation of endothelial cells. In accordance with our observations, Wei et al. (2019) reported that fucoidan extracts with a higher sulfate content had higher bioactivity [32]. Further, a study was published investigating the effect of enzymatic hydrolysis on this, and this present study used high molecular weight (HMW) fucoidan to study bioactivity. Low molecular weight (LMW) and middle molecular weight (MMW) fucoidans had no enhanced anti-angiogenic effect on OEC-MSC-co-cultures. Treatment with MMW fucoidan caused an inflammatory activation in endothelial cells indicated by increased IL-6 and ICAM-1 levels [33].

In the OEC mono-culture system, we observed that treatment with FE_F3 can limit endothelial activation exposed to LPS, which was implied by reduced gene expression of NF-kB, TLR4, IL-6, and adhesion molecules (Figure 2). Further, protein levels of IL-6 and ICAM-1 decreased (Figure 3). On the protein level, this effect was less visible when all endothelial donors were considered as average values. However, when considering the donors individually, a notable decrease was observed for all tested donors.

Many reports indicate the anti-inflammatory properties of fucoidan. However, it is well known that the biological effect of fucoidan varies greatly depending on species and chemical structure [34]. We tested an HMW fucoidan ranging from 110 to 800 kDa, composed of 88% fucose and 9% galactose with a high sulfate content of 39% and negligible protein content (≤0.15%) [26]. Fucoidan derived from *Cladosiphon okamuranus* Tokida with higher uronic acid content has been reported to down-regulate IL-6 production and NF-kB nuclear translocation in an LPS-stimulated epithelial cell line [35]. A high sulfate, low protein fucoidan from *Sargassum swartzii* was extracted enzymatically and subjected to LPS-stimulated RAW 264.7 macrophages. Through its action on TLR-mediated MyD88, IKK complex, it attenuated LPS-induced inflammation inhibiting NF-kB and MAPK activation [36]. Fucoidan, with a molecular weight of 735 kDa obtained by enzyme-assisted extraction from *Fucus vesiculosus* has shown anti-inflammatory activity in a human mononuclear cell line. This was evidenced by an inhibition of the enzymes cyclooxygenase-2 and hyaluronidase. In addition, this extract inhibited the MAPK signaling pathway and exhibited possible anti-hyperglycemic and anti-coagulant activity [37]. Jin and colleagues (2015) observed that fucoidan derived from *Undaria pinnatifida* enhanced the production of pro-inflammatory cytokines IL-6, IL-8, and TNF-α in neutrophils by activating the PI3K/AKT signaling pathway [38]. In contrast, an LMW fucoidan obtained by hydrolysis of an *Undaria pinnatifida* extract inhibited p38 MAPK, ERK, and JNK activity in LPS-stimulated RAW 264.7 macrophages resulting in alleviation of inflammation [39]. This contradictory finding indicates that sugar composition, sulfate content, and molecular weight are important determents of bioactivity [40]. Another approach to understanding the anti-inflammatory bioactivity of fucoidan is to consider the role of TLR4. It is known that monocytes and macrophages develop tolerance to LPS by down-regulating TLR4, leading to a decrease in inflammatory cytokine production [41]. In the present study, we observed a reduced gene expression of TLR4 after fucoidan treatment which may make these cells less susceptible to LPS. A recent study proposed that fucoidan isolated from *Padina commersonii* reduced inflammatory activation of LPS-stimulated RAW 264.7 macrophages by blocking TLR/MyD88/NF-kB signal transduction [42]. Pei et al. (2007) identified fucoidan as a scavenger receptor inhibitor that impaired the internalization of LPS in microglia [43].

Upon initiating the tethering of leukocytes on the endothelium, selectins are a key regulator of leukocyte migration and therefore represent an important target for acute or chronic inflammation [44]. After we observed decreased expression of adhesion molecules P-Selectin and E-Selectin in the OEC mono-cultures by FE_F3 treatment, the functional consequences were verified in the co-culture system. We demonstrated that treatment with fucoidan decreased the adhesion of monocytes to the endothelial monolayer. This was indicated by reduced gene expression of the monocyte surface markers (Figure 4) and further verified by a decrease in observed adhesion using immunofluorescence (Figure 5). This could be caused by the inhibition of NF-kB-activation and corresponding downstream events or by a direct interaction between selectins and fucoidan. Supporting the second assumption, Zhang et al. (2001) hypothesized that fucoidan acts as a ligand to selectin as they observed inhibition of leukocyte recruitment in experimental colitis in mice [45]. Bachelet et al. (2009) used SELDI-TOF mass spectroscopy to detect the binding of an LMW fucoidan extract to P-Selectin. Further, using flow cytometry, they observed the binding of LMW fucoidan to activated human platelets [46]. In addition, Rouzed and colleagues (2011) succeeded in radiolabeling fucoidan to show its binding affinity to P-Selectin, suggesting that ^99m^Tc-fucoidan may be a relevant imaging agent for detecting P-Selectin overexpression in vivo [47]. An LMW fucoidan derived from *Saccharina Japonica* exhibits a high affinity for P-Selectin. In a mouse abdominal aortic aneurysm model, it reduced macrophage infiltration and expression of inflammatory mediators [48].

## 4. Materials and Methods

### 4.1. Extraction of Fucoidan from Algae

*Fucus distichus* subsp. *evanescens* was harvested at Kiel Kanal, Germany, and kindly provided by Costal Research and Management GmbH. Extracts from *Fucus distichus* subsp. *evanescens* were obtained using an enzyme-assisted technique as described previously [26]. In brief, brown algae were treated with Cellic^®^CTec2 cellulase (Novozymes, Bagsværd, Denmark) and alginate lyase SALy from *Sphingomonas* sp. Non-fucoidan polysaccharides were removed by Ca^2+^ and ethanol precipitation, and ion exchange chromatography was performed to yield three pure fractions from the crude fucoidan (FE_F1, FE_F2, and FE_F3). Chemical analyses of the fucoidan fractions were performed and described in [26].

### 4.2. Isolation of Endothelial Progenitor Cells and Outgrowth Endothelial Cells (OECs) from the Peripheral Blood

The isolation of primary cells from human peripheral blood was performed with the approval of the local ethical advisory board of the Medical Faculty of Christian Albrechts University in Kiel and had the consent of the individual donors.

The isolation of endothelial progenitor cells (EPCs) and outgrowth endothelial cells (OECs) was performed as previously described [49]. Briefly, mononuclear cells (MNCs) were isolated from buffy coats by gradient centrifugation using Biocoll (Biochrom, Berlin, Germany). MNCs were resuspended in Endothelial Growth Medium 2 (EGM-2) (Promocell, Heidelberg, Germany), including all associated supplements (Promocell), 5% FBS (Sigma Aldrich, St. Louis, MO, USA), and 1% Penicillin/Streptomycin (PS) (Biochrom) and seeded in collagen type I-coated (Corning, Bedford, MA, USA) 24-well plates at a density of 5 × 10^6^ cells/cm^2^. After one week, mixed cell populations, referred to as endothelial progenitor cells, were harvested for isolation of CD14 positive monocytes or sub-cultured on new collagen-I coated 24-well plates at a density of 5 × 10^5^ cells/cm^2^ to isolate OECs, respectively.

Cobblestone-shaped colonies of OECs grew within two to three weeks after reseeding EPCs. Obtained OECs were cultivated in fibronectin-coated (Millipore, Temecula, CA, USA) plates or flasks in EGM-2 supplemented by 5% FBS and 1% PS. The medium was exchanged every second day, and cells were sub-cultured every three to four days when confluent.

### 4.3. Magnetic Cell Sorting to Isolate Cd14 Positive Monocytes from Human Peripheral Blood

CD14-positive monocytes were isolated either from pre-cultured EPCs gained from buffy coats or directly from fresh peripheral blood using CD14 Dynabeads™ (Invitrogen, Waltham, MA, USA) following the manufacturer’s protocol.

For isolation, EPCs were prepared in an isolation buffer at a density of 1 × 10^7^ cells/mL. Samples from human peripheral blood were prepared by washing steps using an isolation buffer. After adding the magnetic beads and twenty minutes of incubation at 5 °C, tubes were placed in the magnet for two minutes, and the supernatant was discarded, followed by five washing cycles of two minutes. The cell pellets were resuspended in EGM-2, and these cells were prepared for further applications as indicated for the individual methods.

### 4.4. Fucoidan and LPS Treatment of OEC Mono-Cultures and OECMonocyte Co-Cultures

**Figure 6:**
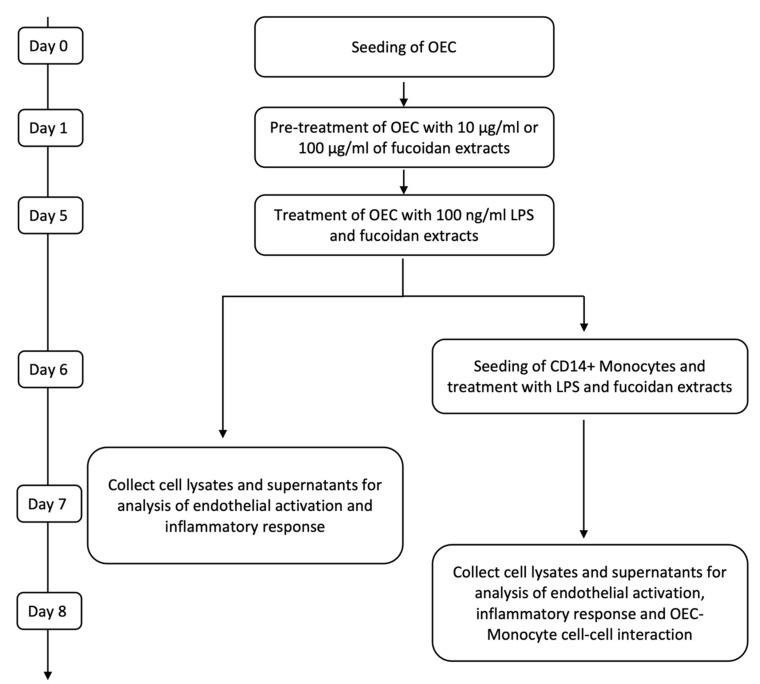
Time scales for treatment and sample harvesting for OEC mono-cultures and co-cultures with monocytes.

OECs were seeded in fibronectin-coated 24-well plates at a density of 52 000 cells/cm^2^. One day after seeding, the medium was refreshed, and 10 µg/mL and 100 µg/mL of fucoidan (FV_crude (Sigma Aldrich), FE_F3) were added. The medium was changed on the third day of treatment. After five days of fucoidan treatment, cells were treated with EGM-2 medium containing 100 ng/mL lipopolysaccharide (LPS) (Sigma Aldrich) in addition to 10 µg/mL or 100 µg/mL of FV_crude or FE_F3. Control cells were cultured in an EGM-2 medium (Figure 6). All experiments were performed with cells from three individual donors. Passage numbers of OECs ranged from 4 to 8.

For co-culture experiments, OECs were seeded and cultured as described above. After pre-treatment with fucoidan on day six, the medium was replaced with fresh medium containing 100 ng/mL LPS and the same fucoidan concentrations described previously. On the next day, CD14+ Monocytes isolated from EPC cultures were added to the OEC mono-culture at a ratio of 20%, and LPS and fucoidan treatment were continued for two days. To verify results gained with CD14+ cells from EPC cultures, selected experiments were performed using co-cultures with CD14+ cells isolated from fresh blood samples using the same experimental procedures (Figure 6).

All experiments were performed with cells from three different donors. Passage numbers of OECs ranged from 4 to 9.

### 4.5. Analysis of Endothelial Cell Activation and Inflammatory Response in Mono- and Co-Cultures by Semiquantative Real-Time PCR

RNA was isolated from OECs in mono-cultures and co-cultures of OECs with EPC-derived CD14-positive monocytes after treatment with fucoidan and LPS at indicated time points (see Figure 6).

To isolate RNA, cell lysis buffer T was applied for ten minutes at 37 °C. RNA isolation was performed using a peqGOLD total RNA kit (PEQLAB, Erlangen, Germany) following the manufacturer’s protocol, including DNA digestion via DNase I (PEQLAB). The RNA concentration was determined photometrically with a NanoDrop (Thermo Fisher, Waltham, MA, USA). To synthesize cDNA from total RNA, a high-capacity RNA-to-cDNA™ kit (Applied Biosystems, Waltham, MA, USA) was used. After the transcription of 1 µg of total RNA, quantitative real-time PCR was performed using a total volume of 20 µL for each reaction. The Mastermix, containing 10 µL SYBR™ select master mix (Applied Biosystems), 2 µL Quantitect Primer (Qiagen, Hilden, Germany, see Table 1), and 4.8 µL nuclease-free water, was mixed with 3.2 µL cDNA. RPL13A was used as the housekeeping gene. For DNA amplification, a two-step program (heating to 50 °C for 2 min, heating to 95 °C for 2 min, 40 cycles of 95 °C for 15 s and 60 °C for 60 s) was applied. Relative gene expression was calculated with the ∆∆cT method. Fucoidan and LPS-treated groups were normalized to the untreated control cells. The plots show the mean values ± SD using three different OEC and monocyte donors.

### 4.6. Enzyme-Linked Immunosorbent Assay (ELISA) for Inflammatory Markers

Supernatants from OEC mono-cultures were collected as indicated in Figure 6 and analyzed for protein levels of IL-6 and soluble ICAM-1 using DuoSet^®^ ELISA (R&D Systems) according to the manufacturer’s protocol. The protein levels were normalized to the control and plotted as means ± SD for three individual donors.

### 4.7. Immunocytochemistry of Endothelial Cell Activation and Interaction with Mononuclear Cells

Co-cultures of OECs with EPC-derived CD14-positive monocytes were seeded on fibronectin-coated µ-slide 8 well plates (ibidi, Gräfelfing, Germany) using the same experimental parameters and treated with fucoidan and LPS as previously described (see Section 4.4). After treatment, cells were washed with PBS and fixed using 4% paraformaldehyde for fifteen minutes, followed by three washing cycles of five minutes. For permeabilization, 0.5% Triton^TM^-X100 (Sigma Aldrich) was added for fifteen minutes. Cells were incubated with 1% BSA in PBS for thirty minutes to block unspecific binding sites. After three washing cycles of five minutes with PBS, the primary antibody was applied for two hours at room temperature. Human CD68 (MAB20401 R&D Systems) was used as a primary antibody. Antibodies were diluted in 1% BSA/PBS according to the manufacturer’s specifications. Cells were washed three times and then incubated with the secondary antibody Alexa 555 (A31087 Invitrogen) at a concentration of 2 µg/mL for one hour at room temperature. For nuclear staining, 2 µg/mL Hoechst 33528 was applied for eight minutes. After several washing steps, wells were mounted using ibidi Mounting Medium (ibidi) and imaged with the Evos FL Auto 2 fluorescence microscope (Thermo Fisher Scientific).

### 4.8. Quantification of Mononuclear Cell Adhesion on Endothelial Cells in Response to Fucoidan

To quantify the effect of fucoidan on the adhesion of native CD14-positive monocytes to the LPS-stimulated endothelial cell layer, monocytes prelabeled with CellTracker™ Green CMFDA (Thermo Fisher) were applied. For this purpose, CD14-positive monocytes, isolated directly from peripheral blood as described above, were prelabeled with 10 µM CMFDA Celltracker for 30 min at 37 °C according to the manufacturer’s protocol. Cells were added to pre-treated endothelial cells in co-cultures as described above (see Section 4.4). After four hours, co-cultures were fixed, and Hoechst 33528 for nuclear counterstaining was applied. For quantification, five frames of each condition were taken at a 20× magnification. Viable monocytes were counted using the software ImageJ Version 1.52q. All experiments were performed with cells from three individual donors. Adherent monocytes were presented relative to the control.

### 4.9. Statistical Analysis

The mean values ± standard deviation (SD) from three individual donors were presented. The statistical significance of the results was calculated with the one-way ANOVA using Graphpad Prism 7.03. Values were considered statistically significant when *p* < 0.05.

## 5. Conclusions

In conclusion, this study investigates the anti-inflammatory properties of the specified and purified fucoidan FE_F3 fraction in an LPS-induced inflammation model using human blood-derived endothelial cells and their co-cultures with MNCs.

In these models, the FE_F3 fraction showed multifactorial influences on a series of pro-inflammatory key molecules and processes. In combination with the anti-angiogenic effects shown in our previous study [18], these present data underline that the FE_F3 fraction is, in particular, qualified to control and lower inflammatory processes mediated by the endothelium. Thus, this fraction offers a broad spectrum of biomedical applications.

## Figures and Tables

**Table 1:** QuantiTect Primer Assays used in the presented study.

Gene	Catalog Number
Interleukin-6 (IL-6)	QT00083720
Nuclear Factor NF-kappa-B p105 subunit (NF-kB)	QT00063791
Toll-like receptor 4 (TLR4)	QT01670123
Glycogen synthase kinase 3 (GSK3β)	QT00057134
Intercellular Adhesion Molecule 1 (ICAM-1)	QT00074900
Vascular Cell Adhesion Protein 1 (VCAM-1)	QT00018347
E-Selectin	QT00015358
P-Selectin	QT00012516
Integrin α 1 (CD11b)	QT00031500
Macrosialin (CD68)	QT00037184
CD163	QT00074641
Mannose receptor C type 1 (CD206)	QT00012810
60S Ribosomal Protein L13a (RPL13A)	QT00089915

## Data Availability

The data presented in this study are available on request from the corresponding author.
